# BEYOND STEP COUNTING: SENSOR-BASED EVALUATION OF HIP FRACTURE PATIENTS’ MOBILITY IN THE REAL WORLD

**DOI:** 10.1093/geroni/igad104.1334

**Published:** 2023-12-21

**Authors:** Carl-Philipp Jansen, Jochen Klenk, Hubert Blain, Beatrix Vereijken, Jorunn Helbostad, Clemens Becker

**Affiliations:** Robert-Bosch-Hospital Stuttgart, Stuttgart, Baden-Wurttemberg, Germany; Robert-Bosch-Hospital Stuttgart, Stuttgart, Baden-Wurttemberg, Germany; Centre Hospitalier Universitaire de Montpellier, Montpellier, Aquitaine, France; Norwegian University of Science and Technology, Trondheim, Nord-Trondelag, Norway; Norwegian University of Science and Technology, Trondheim, Nord-Trondelag, Norway; Robert-Bosch-Hospital Stuttgart, Stuttgart, Baden-Wurttemberg, Germany

## Abstract

After a hip fracture, rehabilitation starts with the primary aim to get older persons back on their feet and mobile again. Multidisciplinary hospital treatment is suggested as the first essential step for optimization of care immediately after the fracture, followed by subacute exercise interventions to further improve mobility. However, reported outcomes of rehabilitation vary widely geographically and within regions and most often consist of repeated physical capacity measures without knowledge of the actual mobility behaviour of patients after hospital care. Evidence emerges that small changes in physical capacity (e.g., gait speed) coincide with larger changes in physical activity (e.g., number of steps) during the first six months post-fracture. In a pooled longitudinal analysis of n=785 hip fracture patients (mean age: 83.4 years, SD=6.1) from Germany and Norway, we found daily walking duration trajectories to increase until week 28 post surgery, and that 50% of the peak mobility was achieved within the first four weeks. Still, in order to better understand the underlying mechanisms of mobility performance and to improve rehabilitation measures, in-depth data on patients’ real-world mobility beyond walking volume is needed. Therefore, in the Mobilise-D project, a novel sensor algorithm is used to quantify patients’ real-world mobility with higher granularity and more extensive information. Baseline data on physical capacity measures, sensor-based mobility outcomes, and patient-reported outcomes of mobility and function of n= 454 (mean age: 78.1 years, SD=9.2) hip fracture patients is presented.

